# Increased Risk of Carotid Atherosclerosis in Male Patients with Chronic Periodontitis: A Nationwide Population-Based Retrospective Cohort Study

**DOI:** 10.3390/ijerph16152635

**Published:** 2019-07-24

**Authors:** Ching Tong, Yu-Hsun Wang, Yu-Chao Chang

**Affiliations:** 1 School of Dentistry, Chung Shan Medical University, Taichung 40201, Taiwan; 2 Division of Endodontics & Periodontology, Department of Stomatology, Taichung Veterans General Hospital, Taichung 40705, Taiwan; 3 Department of Medical Research, Chung Shan Medical University Hospital, Taichung 40201, Taiwan; 4 Department of Dentistry, Chung Shan Medical University Hospital, Taichung 40201, Taiwan

**Keywords:** carotid atherosclerosis, chronic periodontitis, men, nationwide population, cohort study, Taiwan

## Abstract

Carotid artery stenosis is a narrowing or constriction of any part of the carotid arteries, usually caused by atherosclerosis. However, no studies have specifically evaluated the association between carotid atherosclerosis (CA) and chronic periodontitis (CP). This study was to investigate the role of CP in increasing the subsequent risk of CA in the overall Taiwanese population. We carried out this retrospective cohort study, employing data derived from the National Health Insurance Research Database. A total of 72,630 patients who were newly diagnosed with CP from 2001 to 2012 were selected. For a propensity-matched control group, 72,630 healthy patients without CP were picked at random, matched according to age, sex, and index year from the general population. Cox proportional hazard regression analysis, which included sex, age, and comorbidities, was adopted to assess the hazard ratio (HR) of CA between the CP cohort and non-CP cohort. The average ages of the CP and non-CP groups were 44.02 ± 14.63 years and 44.15 ± 14.41 years, respectively. The follow up durations were 8.65 and 8.59 years for CP and non-CP groups, respectively. The results demonstrated that 305 and 284 patients with newly diagnosed CA were found in the CP cohort and non-CP cohort, respectively. There was no significant difference of developing CA in the CP cohort compared with the non-CP cohort (adjusted HR) 1.01, 95% confidence interval (CI, 0.86–1.19). However, multivariate Cox regression analysis indicated that the male group had significantly higher incidence risk of CA (log rank *p* = 0.046). In conclusion, this nationwide retrospective cohort study indicated that male patients with CP exhibited a significantly higher risk of CA than those without CP.

## 1. Introduction

Chronic periodontitis (CP) is a chronic infectious inflammatory disease destroying the periodontium, which may lead to tooth loss [1]. The pathogenesis of CP involves the inflammatory and immunological processes’ response elicited by periodontal pathogens. A growing body of scientific evidence suggests an association between CP and some systemic diseases, such as cardiovascular diseases [2,3], diabetes [4], and respiratory diseases [5]. Because periodontitis may be at a higher risk for many other systemic conditions, good periodontal health is an important component of general health. Dental professionals may need to assume a larger responsibility for the overall health of patients.

Carotid artery stenosis is a narrowing of the large arteries located on each side of the neck that carry blood to the head, face and brain. The carotid artery forks into the internal carotid artery and the external carotid artery at the throat. The fork is a common site for atherosclerosis, an inflammatory build-up of atherosclerotic plaques within the arteries that causes them to narrow [6]. Carotid atherosclerosis (CA) can worsen over time to completely block the artery which may lead to stroke [7]. Therefore, CA could be recognized as a crucial systemic condition.

Studies have clearly demonstrated that CA was associated with CP by systematic review and meta-analysis [8,9]. Previously, periodontal pathogens have been identified in carotid atherosclerotic plaques [3,10,11]. Consistently, patients with atherosclerotic plaques had the highest percentage of severe periodontitis [12]. In addition, the presence of *Porphyromonas gingivalis* and *Prevotella nigrescens* in subgingival lesions was significantly associated with increased CA in a cross-section study [13]. These results suggested that periodontal disease might be a risk indicator for CA.

However, the association between CP and CA still requires further investigations, such as cohort design and relatively large sample size. In this study, a nationwide population-based retrospective cohort study was used to investigate the possible link between CP and CA from the Taiwanese National Health Insurance Research Database (NHIRD).

## 2. Materials and Methods

### 2.1. Data Source

Almost 100% of the Taiwanese population was enrolled in this compulsory National Health Insurance program in 2014 [14]. The Longitudinal Health Insurance Database 2010 (LHID2010) was selected for this cohort study. The registration information and dental and medical data were included in LHID2010, which contains one million beneficiaries randomly sampled from the 2010 registry of beneficiaries in NHIRD. LHID2010 offers encrypted patient identification number, date of birth, sex, diagnostic codes, and the date of visit to medical institutes as described previously [15,16]. This study was approved by the Chung Shan Medical University Hospital institutional review board (CS2-15071).

### 2.2. Exposure of CP

**Figure 1:**
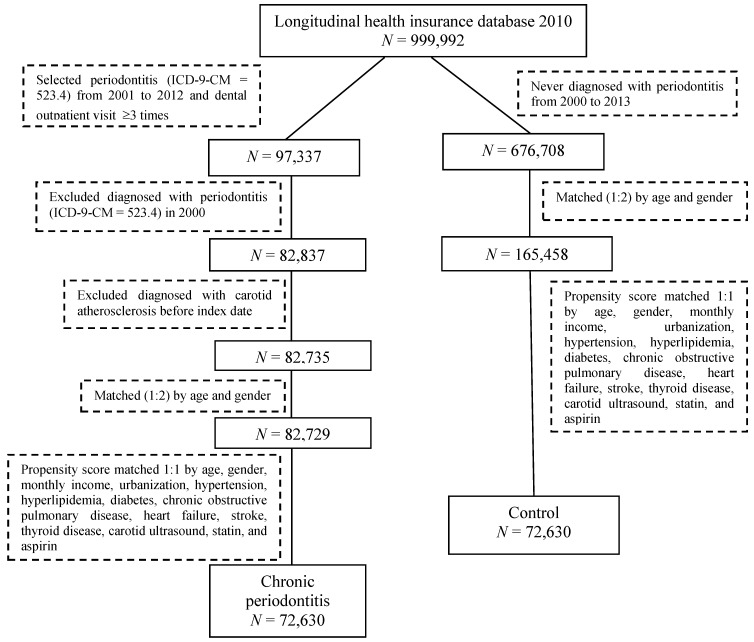
Flow chart of study selection procedure.

This retrospective cohort study enrolled ambulatory patients for a dental visit with newly diagnosed CP and a matched non-CP as a healthy control. The International Classification of Disease, Ninth Revision, Clinical Modification (ICD-9-CM) code 523.4 was used to identify the patients with CP. Patients with newly diagnosed CP from 2001 to 2012 were used as the CP cohort, with the first time of CP diagnosis being defined as the index date. Only patients with at least three outpatient service claims were selected to ensure the accuracy of CP diagnosis. A comparison cohort, the non-CP group, was randomly selected from 2000 to 2013 from participants who were never diagnosed with CP. The controls were then frequency matched according to age, sex, and index year at a 1:2 ratio. Moreover, propensity score matching to select controls was used to reduce the confounding bias. Controls and CP exposure subjects were 1:1 matched using a propensity score by age, sex, monthly income, urbanization, and co-morbidities at the baseline. Patients diagnosed with any type of periodontal diseases before 2001 were excluded. The flow chart of case selection and exclusion is shown in Figure 1.

### 2.3. CA Event

In this population-based, observational cohort study, ICD-9-CM code 433.1 “occlusion and stenosis of carotid artery” was used to identify patients with CA. Patients with newly diagnosed CA from January 2001 to December 2012 were recruited. They were included not only with clinical diagnosis, but also had received outpatient visits ≥3 times or admission ≥1 time to ensure the accuracy of diagnosis. Also, patients with CA were not captured from the index date to the date of the primary outcome, withdrawal from the NHI system or the end of 2013, whichever came first. In order to prevent subclinical carotid disease found at preventive health checkups from contributing to CA, preventive carotid ultrasound examinations (NHI codes: 20013A, 20013B, and 20013C) and secondary prevention drugs with statins or aspirin longer than 30 days were added to propensity matching.

### 2.4. Co-Morbidities

Potential confounders, such as chronic pulmonary disease (ICD-9: 490–496), diabetes (ICD-9: 250), hypertension (ICD-9: 401–405), hyperlipidemia (ICD-9: 272), heart failure (ICD-9: 428), stroke (ICD-9: 430–438), and thyroid disease (ICD-9: 240–246), were considered as potential comorbidities in this study. To improve the validity, these co-morbidities were identified as ≥3 outpatient visits or at least 1 admission within 1 year before the index date.

### 2.5. Statistical Analysis

The Student’s *t* test and chi-square test were used to compare the demographic and clinical characteristics of patients with CP and those without CP. Cox proportional hazard models were applied to estimate the hazard ratios and 95% confidence intervals of CP. The log-rank test was used to compare differences between CP and non-CP cohorts. All statistical analyses were performed with SPSS version 18 (SPSS, Chicago, IL, USA). The level of statistical significance was set at *p* < 0.05.

## 3. Results

The baseline demographic data of this study are listed in Table 1. A total of 72,630 CP patients and 72,630 individuals without CP with similar age, income, and urbanization distributions were selected in this study (*p* > 0.05). There were no significant potential comorbidities differences between CP and the healthy controls (*p* > 0.05).

A total of 305 and 284 patients were newly diagnosed CA in the CP group and non-CP group, respectively. As shown in Table 2, there was no significant difference of developing CA in the CP cohort compared with the non-CP cohort (adjusted hazard ratio (HR) 1.02, 95% confidence interval (CI) 0.86–1.19). The male group had a higher risk of CA than the female group (adjusted HR: 1.30; 95% CI 1.10–1.54). The age groups 40–64 years old (8.72, 95% CI 5.56–13.68) and ≥65 years old (17.10, 95% CI 10.63–27.53) had higher risk compared with the <40 years old group. In the adjusted model, there was no significant risk for CA after controlling for monthly income and urbanization. However, patients with hypertension, hyperlipidemia, diabetes, COPD, stroke, and carotid ultrasound demonstrated significant risk for CA.

**Figure 2:**
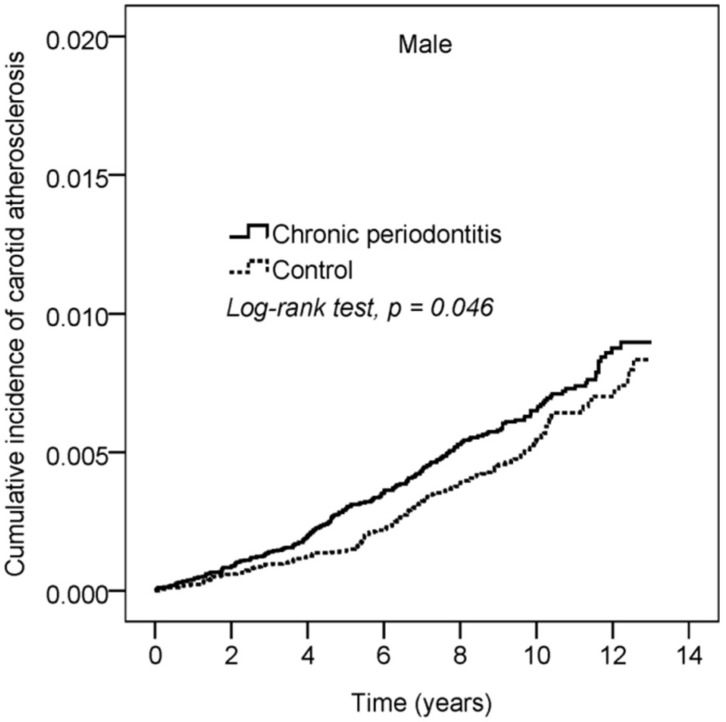
Cumulative incidence of carotid atherosclerosis (CA) in chronic periodontitis (CP) and control groups.

The cumulative incidence curve of CA is shown in Figure 2. After 13 years follow-up, the cumulative incidence of CA in male patients with CP was higher than that in the control subjects (log rank test, *p* = 0.046).

In Table 3, the subgroup analysis of hazard ratios by age revealed that there was no significant difference between the CP cohort and control. In addition, there was no significant difference for female patients with CA between CP cohort and control (HR 0.81, 95% CI 0.63–1.05). Male patients with CA had a 1.16-fold chance of being in the CP-cohort than control cohort (0.94–1.43). For gender difference, *p* for interaction was 0.042.

As shown in Table 4, the mean follow-up durations in the CP and non-CP cohorts were 8.65 years and 8.59 years, respectively. The mean interquartile ranges for CA were 5.69 and 6.07 years for the CP and non-CP cohorts, respectively.

## 4. Discussion

To the best of our knowledge, this is the first nationwide population-based cohort study to report the gender difference in the relationship between CP exposure and CA risk. The risk of CA in male patients with CP exposure was higher than in the non-CP group. Similar results were found by Desvarieux et al. [17] who reported that periodontitis is related to atherosclerosis in men but not women in a Germany cross-sectional population-based survey. Consistently, a cross-sectional study randomly selected from the Swedish civil registration database revealed a significant association between periodontitis and carotid calcification, especially in the older population and men [18]. Taken together, our data provide evidence that male patients with CP are at increasing risk of CA. A regular oral check-up for periodontal status may be necessary for CA patients.

CP may influence CA by an inflammatory build-up of atherosclerotic plaques within carotid arteries that causes them to narrow. Mechanisms underlying this association are not clear. Several factors have been considered, including periodontal pathogens that can infiltrate periodontitis-associated atherosclerotic plaques and be detected by polymerase chain reaction [3,10,11]. This may be a direct association between the periodontal infection and the pathophysiology of CA. The bacterial pathogens derived from the subgingival biofilm might be directly or indirectly involved in the process of atherogenesis.

Atherosclerosis is recognized as a chronic inflammatory disease of the arterial wall. Periodontal disease is a chronic inflammatory disease characterized by the destruction of supportive tissues of teeth in response to infection with various Gram-negative anaerobes. Therefore, low-grade inflammation of CP may play a role in the pathogenesis/progression of CA.

Systemic inflammation might cause an arterial hemodynamic derangement when patients suffer from periodontal disease. Claudio et al. [19] have reported that worse periodontal health was related with the presence of CA. Periodontal inflammation is associated with the increased parietal tension in the carotid artery via echo-Doppler examination [20]. Taken together, patients with CP may alter the hemodynamic pattern then leading to CA.

However, some potential limitations should be addressed. First, because many patients with CA are asymptomatic [7], the diagnosis of CA might be underestimated with the ICD-9 code in this study. Second, clinical data, such as carotid intima-media thickness, arterial diameter, and blood flow velocity, are unavailable in the claims database. As atherosclerosis is a systemic disease, we further added the carotid ultrasound examination and prevention drugs for propensity matching to solve this systemic condition. Third, the severity of CA, the severity of periodontal status, body weight, smoking habits, alcohol use, family history or nutrition status were not available in LHID2010 to investigate the contributions of these factors. Four, the use of dental care was estimated to be up to 45% of Taiwan’s population in 2013 [14]. The prevalence of CP might be underestimated from the NHIRD. In addition, unrecognized CP might be included in the healthy controls within this register-based study. However, the use of a nationwide population-based database can provide sufficient sample size, generalizability, and statistical power to assess the association between CA and CP in the Taiwanese population.

## 5. Conclusions

In conclusion, this nationwide cohort study indicated an increased risk of CA among CP male patients. The results may assist in treatment strategies for CA of dental and medical experts in the national medical care system. Male patients with CA should receive regular periodontal evaluation. Further prospective studies are needed to evaluate the causal relationship between CA and CP.

## Figures and Tables

**Table 1:** Demographic data of matched study cohorts.

	Chronic Periodontitis (*N* = 72,630)		Control (*N* = 72,630)		*p*-Value
	*n*	%	*n*	%	
Age					0.290
<40	30,585	42.1	30,597	42.1	
40–64	35,039	48.2	35,198	48.5	
≥65	7006	9.6	6835	9.4	
Mean ± SD	44.02 ± 14.63		44.15 ± 14.41		0.107
Gender					0.258
Female	38,252	52.7	38,467	53.0	
Male	34,378	47.3	34,163	47.0	
Monthly income					0.177
<NTD $20,000	31,190	42.9	30,876	42.5	
NTD $20,000–NTD $40,000	22,390	30.8	22,679	31.2	
>NTD $40,000	19,050	26.2	19,075	26.3	
Urbanization					0.803
Urban	49,325	67.9	49,367	68.0	
Suburban	19,208	26.4	19,224	26.5	
Rural	4097	5.6	4039	5.6	
Hypertension	8973	12.4	8985	12.4	0.924
Hyperlipidemia	4390	6.0	4263	5.9	0.159
Diabetes	4002	5.5	3957	5.4	0.604
COPD	3022	4.2	2973	4.1	0.518
Heart failure	347	0.5	287	0.4	0.017
Stroke	1235	1.7	1132	1.6	0.033
Thyroid disease	1325	1.8	1328	1.8	0.953
Carotid ultrasound	5702	7.9	5505	7.6	0.053
Statin	13,206	18.2	13,270	18.3	0.664
Aspirin	10,852	14.9	10,915	15.0	0.643

The Student’s *t* test and chi-squared test were used to test the difference of continuous and categorical variables, respectively. COPD: chronic obstructive pulmonary disease. SD: standard deviation. NTD: New Taiwan Dollars.

**Table 2:** Risk factor analysis of carotid atherosclerosis.

	No. of Event	Observed Person-Years	ID	Crude HR	95% CI	Adjusted HR	95% CI
Chronic periodontitis							
No	284	624,078	0.5	1		1	
Yes	305	628,342	0.5	1.06	0.91–1.25	1.02	0.86–1.19
Age							
<40	21	543,733	0.04	1		1	
40–64	307	596,995	0.5	13.50	8.68–21.00	8.72	5.56–13.68
≥65	261	111,691	2.3	62.41	40.01–97.36	17.10	10.63–27.53
Gender							
Female	237	663,089	0.4	1		1	
Male	352	589,331	0.6	1.67	1.42–1.97	1.37	1.15–1.62
Monthly income							
<NTD $20,000	325	536,670	0.6	1		1	
NTD $20,000–NTD $40,000	167	384,515	0.4	0.72	0.60–0.87	1.03	0.84–1.25
>NTD $40,000	97	331,236	0.3	0.48	0.39–0.61	0.85	0.66–1.09
Urbanization							
Urban	383	850,525	0.5	1		1	
Suburban	170	332,359	0.5	1.14	0.95–1.36	1.15	0.96–1.38
Rural	36	69,536	0.5	1.15	0.82–1.62	0.87	0.61–1.24
Hypertension	266	141,725	1.9	6.68	5.67–7.85	1.72	1.42–2.08
Hyperlipidemia	98	64,594	1.5	3.87	3.11–4.81	1.38	1.09–1.76
Diabetes	111	61,758	1.8	4.64	3.77–5.70	1.47	1.17–1.83
COPD	86	51,674	1.7	3.97	3.16–4.99	1.50	1.18–1.90
Heart failure	11	4737	2.3	5.25	2.89–9.53	1.06	0.58–1.93
Stroke	68	18,367	3.7	9.05	7.03–11.65	1.57	1.20–2.05
Thyroid disease	16	22,481	0.7	1.53	0.93–2.52	1.28	0.77–2.11
Carotid ultrasound	310	103,674	3.0	12.08	10.27–14.2	4.72	3.91–5.69
Statin	206	238,841	0.9	2.25	1.90–2.66	0.63	0.52–0.76
Aspirin	319	199,526	1.6	6.11	5.20–7.19	1.25	1.02–1.53

ID: Incidence density, per 1000 person-years. COPD: chronic obstructive pulmonary disease. HR: hazard ratio. Adjusted for age, gender, monthly income, urbanization, hypertension, hyperlipidemia, diabetes, COPD, heart failure, stroke, thyroid disease, carotid ultrasound, statin, and aspirin.

**Table 3:** Subgroup analysis of hazard ratios of chronic periodontitis.

	Chronic Periodontitis		Control		HR	95% CI
	*N*	No. of Event	*N*	No. of Event		
Age ^†^						
<40	30,585	11	30,597	10	1.06	0.45–2.50
40–64	35,039	156	35,198	151	1.02	0.82–1.28
≥65	7006	138	6835	123	1.02	0.80–1.30
*p* for interaction = 0.985						
Gender ^‡^						
Female	38,252	109	38,467	128	0.81	0.63–1.05
Male	34,378	196	34,163	156	1.16	0.94–1.43
*p* for interaction = 0.042						

^†^ Adjusted for gender, monthly income, hypertension, hyperlipidemia, COPD, carotid ultrasound, statin, and aspirin. ^‡^ Adjusted for age, monthly income, urbanization, hypertension, hyperlipidemia, diabetes, COPD, heart failure, stroke, thyroid disease, carotid ultrasound, statin, and aspirin.

**Table 4:** Track time of chronic periodontitis and control cohort.

	Chronic Periodontitis (*N* = 72,630)	Control (*N* = 72,630)	*p*-Value
Follow-up duration (years)	8.65 ± 3.06	8.59 ± 3.08	<0.001
Time to event (years), *N* = 589	5.69 ± 3.24	6.07 ± 3.14	0.149

The Student’s *t* test was used to test the difference of continuous variables.

## References

[1] Kinane D.F., Bartold P.M. (2007). Clinical relevance of the host responses of periodontitis. Periodontol. 2000.

[2] Beck J., Garcia R., Heiss G., Vokonas P.S., Offenbacher S. (1996). Periodontal disease and cardiovascular disease. J. Periodontol..

[3] Haraszthy V.I., Zambon J.J., Tresian M., Zeid M., Genco R.J. (2000). Identification of periodontal pathogens in atheromatous plaques. J. Periodontol..

[4] Polak D., Shapira L. (2018). An update on the evidence for pathogenic mechanisms that may link periodontitis and diabetes. J. Clin. Periodontol..

[5] Scannapieco F.A., Cantos A. (2016). Oral inflammation and infection, and chronic medical diseases: Implications for the elderly. Periodontol. 2000.

[6] International Carotid Stenting Study investigators (2010). Carotid artery stenting compared with endarterectomy in patients with symptomatic carotid stenosis (International Carotid Stenting Study): An interim analysis of a randomized controlled trial. Lancet.

[7] Naylor A.R., Bown M.J. (2011). Stroke after cardiac surgery and its association with asymptomatic carotid disease: An updated systematic review and meta-analysis. Eur. J. Vasc. Endovasc. Surg..

[8] Orlandi M., Suvan J., Petrie A., Donos N., Masi S., Hingorani A., Deanfield J., D’Aiuto F. (2014). Association between periodontal disease and its treatment, flow-mediated dilatation and carotid intima-media thickness: A systematic review and meta-analysis. Atherosclerosis.

[9] Zeng X.T., Leng W.D., Lam Y.Y., Yan B.P., Wei X.M., Weng H., Kwong J.S. (2016). Periodontal disease and carotid atherosclerosis: A meta-analysis of 17,330 participants. Int. J. Cardiol..

[10] Kozarov E.V., Dorn B.R., Shelburne C.E., Dunn W.A., Progulske-Fox A. (2005). Human atherosclerotic plaque contains viable invasive *Actinobacillus actinomycetemcomitans* and *Porphyromonas gingivalis*. Arteriosclerosis Thromb. Vas. Biol..

[11] Figuero E., Sánchez-Beltrán M., Cuesta-Frechoso S., Tejerina J.M., del Castro J.A., Gutiérrez J.M., Herrera D., Sanz M. (2011). Detection of periodontal bacteria in atheromatous plaque by nested polymerase chain reaction. J. Periodontol..

[12] Pinho M.M., Faria-Almeida R., Azevedo E., Manso M.C., Martins L. (2013). Periodontitis and atherosclerosis: An observational study. J. Periodontal. Res..

[13] Yakob M., Söder B., Meurman J.H., Jogestrand T., Nowak J., Söder P.Ö. (2011). *Prevotella nigrescens* and *Porphyromonas gingivalis* are associated with signs of carotid atherosclerosis in subjects with and without periodontitis. J. Periodontal. Res..

[14] National Health Insurance Administration, Ministry of Health and Welfare, Taiwan, R.O.C. National Health Insurance Annual Report 2014–2015. https://nhird.nhri.org.tw/en/.

[15] Yu H.C., Chen T.P., Wei C.Y., Chang Y.C. (2018). Association between Peptic Ulcer Disease and Periodontitis: A Nationwide Population-Based Case-Control Study in Taiwan. Int. J. Environ. Res. Public Health.

[16] Chen C.K., Huang J.Y., Wu Y.T., Chang Y.C. (2018). Dental Scaling Decreases the Risk of Parkinson’s Disease: A Nationwide Population-Based Nested Case-Control Study. Int. J. Environ. Res. Public Health.

[17] Desvarieux M., Schwahn C., Völzke H., Demmer R.T., Lüdemann J., Kessler C., Jacobs D.R., John U., Kocher T. (2004). Gender differences in the relationship between periodontal disease, tooth loss, and atherosclerosis. Stroke.

[18] Bengtsson V.W., Persson G.R., Berglund J., Renvert S. (2016). A cross-sectional study of the associations between periodontitis and carotid arterial calcifications in an elderly population. Acta Odontol. Scand..

[19] Carallo C., Fortunato L., de Franceschi M.S., Irace C., Tripolino C., Cristofaro M.G., Giudice M., Gnasso A. (2010). Periodontal disease and carotid atherosclerosis: Are hemodynamic forces a link?. Atherosclerosis.

[20] Carallo C., De Franceschi M.S., Tripolino C., Figliuzzi M., Irace C., Fortunato L., Gnasso A. (2013). Common carotid and brachial artery hemodynamic alterations in periodontal disease. J. Clin. Periodontol..

